# Supplementary material for: Posttranscriptional Regulation of 14q32 MicroRNAs by the CIRBP and HADHB during Vascular Regeneration after Ischemia
Source: Mol Ther Nucleic Acids. 2018 Dec 6;14:329–38. doi: 10.1016/j.omtn.2018.11.017 (PMC6350214; doi:10.1016/j.omtn.2018.11.017)
Supplement: Document S1. Figure S1 and Table S1 [file mmc1.pdf]

OMTN, Volume *14*

Supplemental Information

Posttranscriptional Regulation of 14q32 MicroRNAs by the CIRBP and HADHB during Vascular Regeneration after Ischemia

Angela Downie Ruiz Velasco, Sabine M.J. Welten, Eveline A.C. Goossens, Paul H.A. Quax, Juri Rappsilber, Gracjan Michlewski, and A. Yaël Nossent

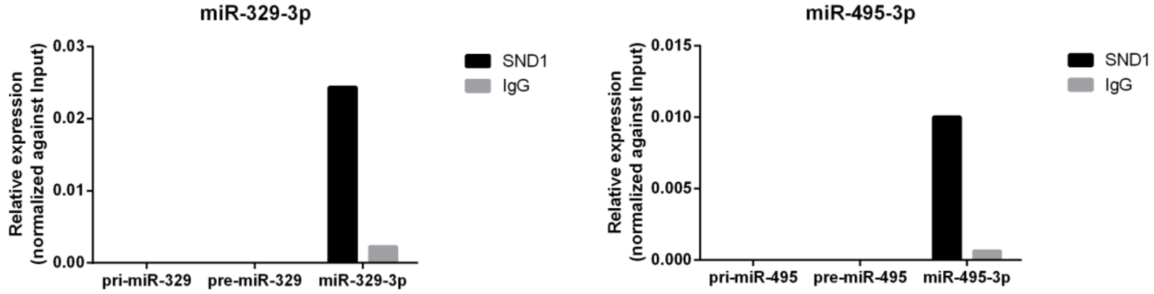

Supplemental Figure 1. RNA binding protein immunoprecipitation with SND1 antibody. Pri-miR, pre-miR and mature microRNA expression levels of miR-329-3p and miR-495-3p were measured in 3T3 cell lysates after immunoprecipitation with SND1 antibody and a non-specific IgG antibody.

Supplementary Table 1. List of primers used.

Gene	Forward Primer	Reverse Primer
primiR-329	AAGGTCACGTTGGGGAATTA	ACCACGAAGCCTCCAAGAT
premiR-329	TGGTACCGGAAGAGAGGTTTT	AGGTTAGCTGGGTGTGTTTCA
primiR-495	AGCATCCCTTCACACTCAGG	GAGCTCTCCAAGGTGAGATTTG
premiR-495	GTTGCCCATGTTATTTTTCG	AGTGCACCATGTTTGTTTCG
CIRBP	TTTTCGTGGGAGGACTCAGC	CCCTGTCCTTTACCACCACC
HADHB	CAGCGCCTGTCCTTACTCAG	CAGAGTGGCCCATGGTCTC
