# Supplementary material for: Posttranscriptional Regulation of 14q32 MicroRNAs by the CIRBP and HADHB during Vascular Regeneration after Ischemia
Source: Mol Ther Nucleic Acids. 2018 Dec 6;14:329–38. doi: 10.1016/j.omtn.2018.11.017 (PMC6350214; doi:10.1016/j.omtn.2018.11.017)
Supplement: Document S2. Article plus Supplemental Information [file mmc2.pdf]

Molecular Therapy
Nucleic Acids
Original Article

Posttranscriptional Regulation of 14q32 MicroRNAs by the CIRBP and HADHB during Vascular Regeneration after Ischemia

Angela Downie Ruiz Velasco,[1,2,8] Sabine M.J. Welten,[3,4,8] Eveline A.C. Goossens,[3,4] Paul H.A. Quax,[3,4] Juri Rappsilber,[2,5] Gracjan Michlewski,[1,2,6,9] and A. Yaël Nossent[3,4,7,9]

[1]Division of Infection and Pathway Medicine, University of Edinburgh, The Chancellor's Building, Edinburgh, UK; [2]The Wellcome Centre for Cell Biology, University of Edinburgh, Edinburgh, UK; [3]Department of Surgery, Leiden University Medical, Leiden, the Netherlands; [4]Einthoven Laboratory for Experimental Vascular Medicine, Leiden University Medical, Leiden, the Netherlands; [5]Department of Biotechnology, Technische Universität Berlin, Berlin, Germany; [6]Zhejiang University – University of Edinburgh Institute, Zhejiang University School of Medicine, Zhejiang University, Haining, Zhejiang, P.R. China; [7]Ludwig Boltzmann Cluster for Cardiovascular Research, Vienna, Austria

After induction of ischemia in mice, 14q32 microRNAs are regulated in three distinct temporal patterns. These expression patterns, as well as basal expression levels, are independent of the microRNA genes' order in the 14q32 locus. This implies that posttranscriptional processing is a major determinant of 14q32 microRNA expression. Therefore, we hypothesized that RNA binding proteins (RBPs) regulate posttranscriptional processing of 14q32, and we aimed to identify these RBPs. To identify proteins responsible for this posttranscriptional regulation, we used RNA pull-down SILAC mass spectrometry (RP-SMS) on selected precursor microRNAs. We observed differential binding of cold-inducible RBP (CIRBP) and hydroxyacyl-CoA dehydrogenase trifunctional multienzyme complex subunit beta (HADHB) to the precursors of late-upregulated miR-329-3p and unaffected miR-495-3p. Immunohistochemical staining confirmed expression of both CIRBP and HADHB in the adductor muscle of mice. Expression of both CIRBP and HADHB was upregulated after hindlimb ischemia in mice. Using RBP immunoprecipitation experiments, we showed specific binding of CIRBP to pre-miR-329 but not to pri-miR-329. Finally, using CRISPR/Cas9, we generated *HADHB*$^{-/-}$ 3T3 cells, which display reduced expression of miR-329 and miR-495 but not their precursors. These data suggest a novel role for CIRBP and HADHB in posttranscriptional regulation of 14q32 microRNAs.

INTRODUCTION

MicroRNAs (miRNAs) are short, non-coding RNA molecules (∼22 nt) that decrease expression of their target genes via translational repression.[1] MicroRNA genes are transcribed by RNA polymerase II as primary microRNA (pri-miRNA) transcripts. Subsequently, the microprocessor complex, containing the RNase III Drosha and co-factor DGCR8, processes these pri-miRNAs into precursor microRNAs (pre-miRNAs) about 70 nt long. Pre-miRNAs are exported to the cytoplasm, where the enzyme Dicer cleaves the pre-miRNA into a microRNA duplex. Generally, one strand of the microRNA duplex (guide strand) is preferred for association with an Argonaute (AGO) protein and loading into the RNA-induced silencing complex (RISC). However, accumulating evidence suggests that the other strand (passenger strand) can also be loaded into the RISC.[2,3] MicroRNA guide the RISC to specific mRNA targets to control mRNA translation.[1] A single microRNA is able to target numerous genes and, by doing so, that microRNA can regulate complex physiological processes. Over the past decade, microRNAs have been shown to play an important role in human disease, including cardiovascular disease.[4–9] Although microRNAs regulate physiological and pathological processes via modulation of target gene expression, microRNA expression itself is also subject to regulation.

MicroRNA expression can be regulated at the transcriptional as well as posttranscriptional level. Processing of microRNA precursors is controlled during the conversion of pri-miRNA to pre-miRNA through modulation of Drosha and DGCR8 activity.[10–17] The conversion of pre-miRNA to mature miRNA is regulated at the level of Dicer cleavage. RNA binding proteins (RBPs) have been found to bind sequences in the terminal loop and stem of pri-miRNAs, enhancing or inhibiting pri- to pre-miRNA cleavage.[10,12,17–19] For example, processing of pri-miRNAs with conserved terminal loop regions, such as pri-miR-18a or pri-let-7a, have been shown to be stimulated and inhibited by heterogeneous nuclear ribonucleoprotein (hnRNP) A1

Received 7 September 2018; accepted 21 November 2018;
https://doi.org/10.1016/j.omtn.2018.11.017.

[8]These authors contributed equally

[9]These authors contributed equally

Correspondence: Gracjan Michlewski, Division of Infection and Pathway Medicine, University of Edinburgh, The Chancellor's Building, Edinburgh, UK.
E-mail: gracjan.michlewski@ed.ac.uk
Correspondence: A. Yaël Nossent, Department of Surgery, Leiden University Medical, Leiden, the Netherlands.
E-mail: a.y.nossent@lumc.nl

This is an open access article under the CC BY license (http://creativecommons.org/licenses/by/4.0/).

protein, respectively.[12,13,20] In addition, p53 and SMADs have been reported to interact indirectly with Drosha and modulate pri- to pre-miRNA cleavage.[10,14] The RBPs LIN28a and LIN28b have been reported to block accumulation of let-7 levels by repression of both Drosha[19,21,22] and Dicer.[21,23–26]

Many microRNAs are encoded within clusters and can be transcribed as long polycistronic transcripts. With 54 microRNA precursors, the 14q32 cluster is the largest polycistronic microRNA gene cluster in humans, to our knowledge. In mice, this cluster is located on chromosome 12F1 and contains 61 microRNA genes. We have previously described the differential regulation of 14q32 microRNAs in a mouse model for ischemia in the hindlimb.[27] Furthermore, we showed that 14q32 microRNA expression can be regulated by the transcription factor MEF2A, not via altered transcription but via direct binding of MEF2A to precursor microRNAs.[28] The microRNAs from the 14q32 cluster follow three different expression patterns after induction of ischemia. Some microRNAs are upregulated early (early-upregulated) and some late (late-upregulated), and the levels of some microRNAs remain unchanged (unaffected). These patterns are independent of the chromosomal location of the 14q32 microRNA genes. Furthermore, the baseline expression levels of these 14q32 microRNAs vary greatly. These findings indicate that individual 14q32 microRNA expression is regulated predominantly at the posttranscriptional level.[27]

We hypothesized that RBPs regulate the posttranscriptional processing of 14q32 microRNAs, and, in this study, we aimed to identify these RBPs. We could show that 14q32 microRNAs are indeed regulated at the posttranscriptional level. We identified two RBPs (cold-inducible RBP [CIRBP] and hydroxyacyl-coenzyme A [CoA] dehydrogenase trifunctional multienzyme complex subunit beta [HADHB]) that bind and aid in the processing of specific 14q32 microRNA precursors. This helps to explain the differential expression of 14q32 microRNAs under ischemia and expands our knowledge of the regulation of microRNA biogenesis under pathological conditions. Because 14q32 microRNAs play a crucial role in post-ischemic neovascularization, understanding how the 14q32 microRNAs are controlled will be relevant to future molecular therapies in cardiovascular disease.

RESULTS

In Vivo MicroRNA Regulation

An microRNA microarray was performed to determine the differential expression of microRNAs after induction of ischemia *in vivo*. MicroRNAs from the 14q32 cluster showed three different temporal expression patterns after single ligation of the femoral artery. One-third of the 14q32 microRNAs were upregulated within 24 hr after ischemia induction (early-upregulated, Figure 1A; average expression, Figure 1B). Another third of 14q32 microRNAs were upregulated 72 hr after induction of ischemia (late-upregulated, Figure 1C; average expression, Figure 1D), whereas the other 14q32 microRNAs were not differentially expressed after ischemia (unaffected, Figure 1E; average expression, Figure 1F). When looking at the distribution of early- and late-upregulated and unaffected microRNAs on the 14q32 locus, there was no association between the expression profiles of microRNAs and their corresponding gene's chromosomal location (Figure 1G). In addition, the baseline expression levels of 14q32 microRNAs were variable and also independent of their corresponding gene's chromosomal location. Because of their proven efficacy in post-ischemic neovascularization, we focused on early-upregulated miR-494-3p, late-upregulated miR-329-3p, and unaffected miR-495-3p for further experiments.[27]

Pri-miRNA, Pre-miRNA, and Mature MicroRNA Levels of 14q32 MicroRNAs

MicroRNAs are transcribed as primary transcripts (pri-miRNAs), which are cleaved into pre-miRs in the nucleus by DROSHA and DCGR8. The pre-miRNAs are then transported into the cytoplasm and cleaved further into mature microRNAs by DICER, potentially facilitated by other RBPs (Figure 2A). Using specific primers for each of the processing steps of three 14q32 microRNAs (miR-329-3p, miR-494, and miR-495-3p), we determined the expression levels of the pri-miRNA, pre-miRNA, and mature microRNAs in adductor muscle tissue after induction of ischemia in mice. Expression of pri-miR-329 decreased slightly after induction of ischemia but increased in expression by day 7 after induction of ischemia. Pre-miR-329 followed expression of pri-miR-329 24 hr after ischemia but continued to decrease until 72 hr after ischemia induction. Expression of mature miR-329-3p showed an opposite trend as pre-miR-329 (Figure 2B). For early-upregulated miR-494, expression of the pri-miRNA transcript was also decreased 24 and 72 hr after ischemia induction. The abundance of pre-miR-494, however, was only slightly reduced 24 hr after ischemia, whereas mature miR-494-3p levels were upregulated (Figure 2C). Expression of pri-miR-495 and pre-miR-495 followed the same pattern after ischemia, showing decreased expression within 24 hr, which continued at later time points (Figure 2D). Despite this, mature miR-495-3p levels remained unchanged after ischemia induction. In general, although pre-miRNA expression levels declined, we observed increased (miR-494 and miR-329) or sustained (miR-495) expression of mature microRNA levels. This suggests that regulation of 14q32 miR-329, miR-494, and miR-495 processing takes place at the conversion of pre-miRNA to mature microRNA.

Pre-miRNA Pull-Down followed by SILAC Mass Spectrometry Reveals Putative 14q32 MicroRNA Biogenesis Factors

We decided to further explore this regulation by comparing the pre-miRNA-interacting protein partners of the upregulated miR-329 and the unaffected miR-495 upon serum starvation in 3T3 cells, mimicking the nutrient depravation during ischemia. We did not pursue miR-494 because of lack of response in our *in vitro* starvation model. To identify the RBPs responsible for differential expression of these two microRNAs via posttranscriptional regulation, we performed RNA pull-down (RP) stable isotope labeling of amino acids (SILAC) mass spectrometry (RP-SMS)[46] (Figures 3A and 3B). We identified a number of proteins that were specifically bound to pre-miR-329 and pre-miR-495 (namely RBM28 and TBL3 [pre-miR-329-specific] and RBM15 [pre-miR-495-specific]) as well as the proteins CKAP4, P4HB, CIRBP, and HADHB, which

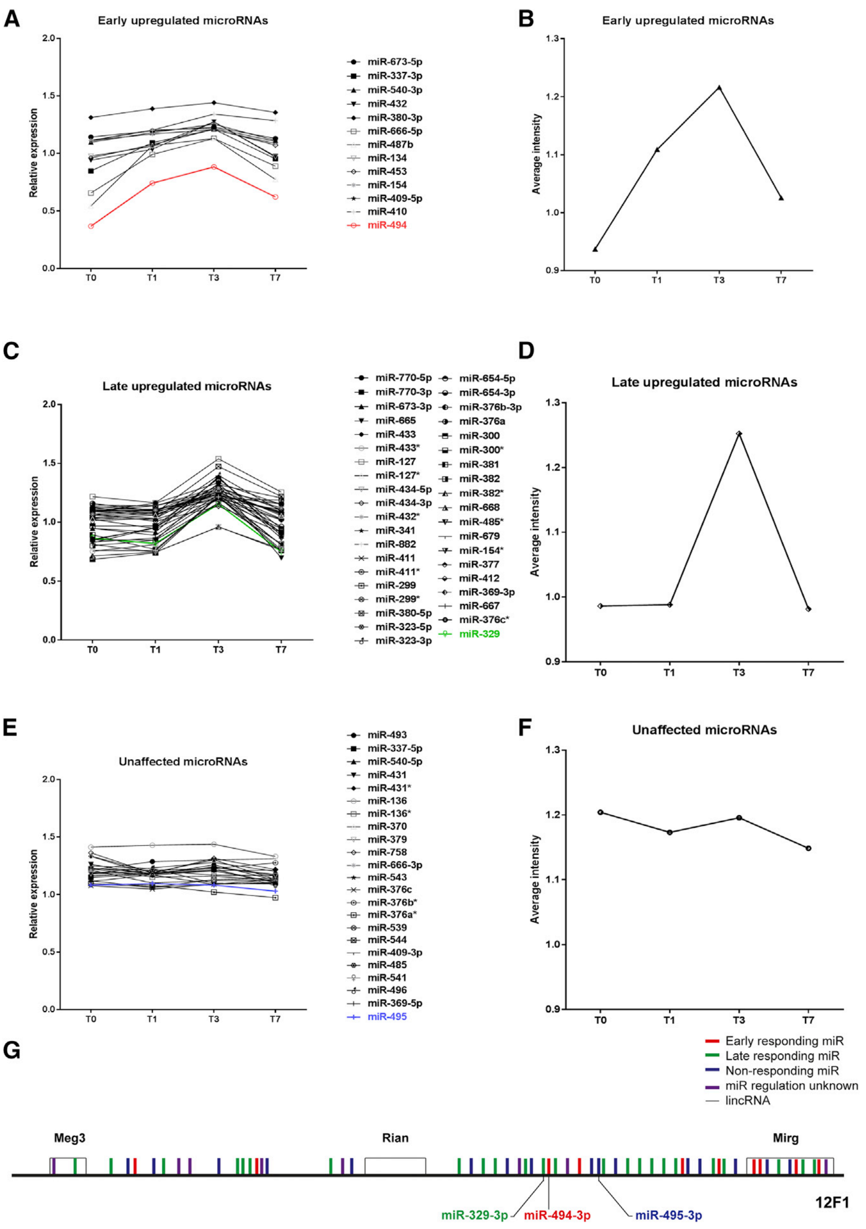

Figure 1. Differential Expression Patterns of 14q32 MicroRNAs after Induction of Ischemia

MicroRNA expression was evaluated before induction of ischemia (T0) and day 1 (T1), day 3 (T3), and day 7 (T7) after induction of ischemia in whole adductor muscle of 4 mice per time point. (A) Early-upregulated 14q32 microRNAs were upregulated within 24 hr after ischemia. (B) Average intensity of all early-upregulated microRNAs. (C) Late-upregulated 14q32 miRs were not upregulated until 72 hr after ischemia induction. (D) Average intensity of all late-upregulated miRs. (E) Unaffected 14q32 microRNAs were not regulated after ischemia. (F) Average intensity of all unaffected miRs. (G) Chromosomal location of early-upregulated (red), late-upregulated (green), and unaffected (blue) microRNAs on the murine 12F1 locus.

were bound to both pre-miR-329 and pre-miR-495, showing an increase in binding after serum starvation. Using western blot analysis, we could only validate binding of HADHB and CIRBP to miR-329 and miR-495 precursors (Figures 3C–3E). This could either be due to the quality of the available antibodies against other identified proteins or due to low expression levels. Although both HADHB and CIRBP showed a general increase in binding upon serum starvation to both pre-miRs, this was particularly evident in the case of HADHB binding to pre-miR-329, which displayed little to no binding under non-starvation conditions but showed an increase in binding upon starvation. A more modest increase in HADHB binding to pre-miR-495 was also observed, where, in contrast to pre-miR-329, this protein also binds under normal serum conditions.

CIRBP and HADHB Expression *In Vivo*

We next determined expression of RBPs CIRBP and HADHB *in vivo* in the adductor muscle at different time points after induction of ischemia. Using immunohistochemistry, expression of both CIRBP and HADHB was confirmed in the adductor muscle of mice on day 1 after ischemia. Expression of CIRBP and HADHB was dispersed throughout the cells (Figures 4A and 4B, respectively). At the mRNA level, the expression of CIRBP was increased within 24 hr after induction of ischemia, whereas HADHB mRNA was increased 3 days after induction of ischemia (Figures 4C and 4D). Importantly, this time point (day 3) corresponds to the maximum changes observed for levels of mature miR-329 as well as pre-miR-329 and pre-miR-495 after induction of ischemia. Next we wanted to confirm a change in protein expression using western blotting. Because of a low signal, CIRBP protein levels could not be detected. Importantly, however, we were able to confirm an increase in HADHB in the adductor muscle on day 3 after induction of ischemia compared with day 0 and day 1 (Figure 4E and 4F), in line with the results obtained for HADHB mRNA.

HADHB and CIRBP Binding to miR-329 Pri-miRNA, Pre-miRNA, and Mature MicroRNA Transcripts

To determine the interaction between HADHB and CIRBP and 14q32 miR-329 and miR-495 precursors, RNA immunoprecipitation (RIP) experiments were performed using antibodies against these RBPs or a negative control immunoglobulin G (IgG) in 3T3 cell cultures. Although HADHB bound to both pri-miR-329 and pre-miR-329, CIRBP showed specific binding to pre-miR-329 but not to pri-miR-329 (Figure 5). CIRBP and HADHB both bound the

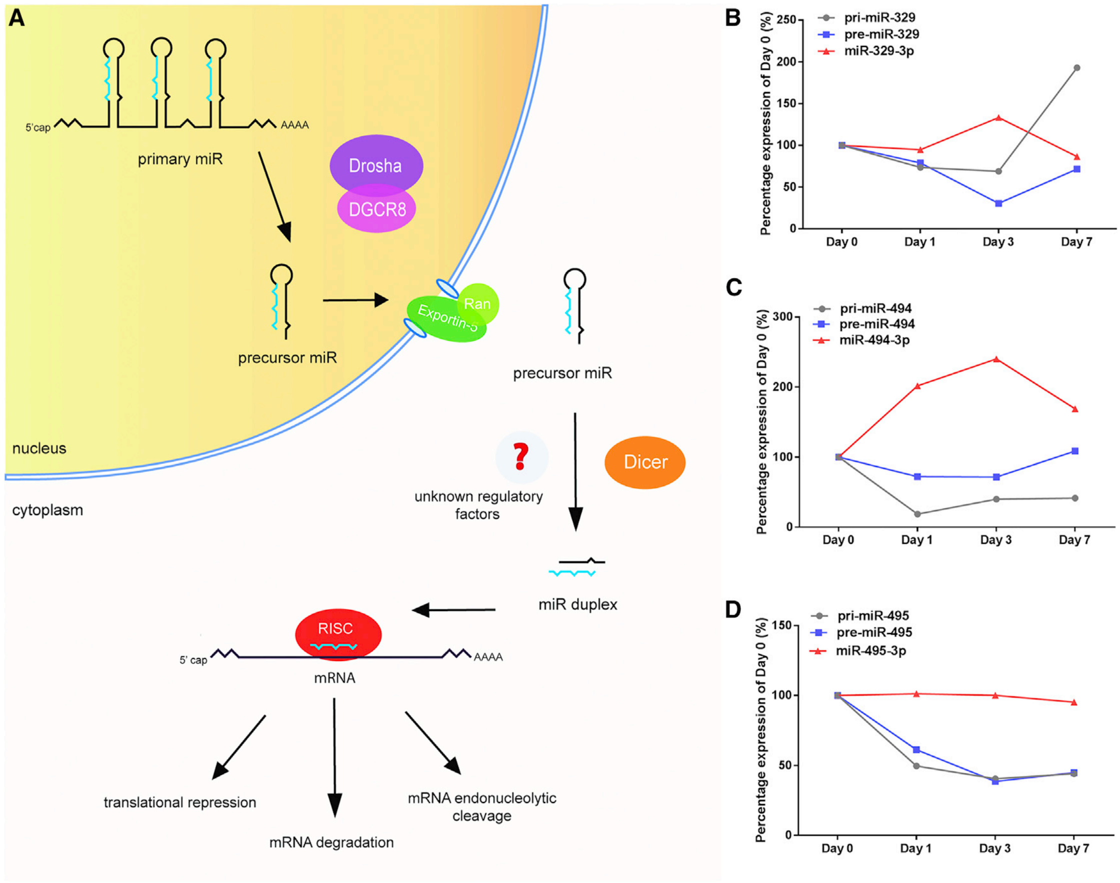

Figure 2. MicroRNA Processing

(A) MicroRNAs are transcribed as primary transcripts (pri-miRNAs), which are cleaved into precursor miRs (pre-miRNAs) inside the nucleus by DROSHA and DCGR8. The pre-miRNAs are then transported into the cytoplasm and cleaved further into mature microRNAs by DICER, potentially facilitated by unknown regulatory factors. (B–D) Pri-miRNA, pre-miRNA, and mature microRNA expression levels of 14q32 microRNAs, measured in whole adductor muscle of 4 mice per time point. Percentage of expression (relative to day 0) on day 0 (no ischemia), day 1, day 3, and day 7 after ischemia induction of pri-miR-329, pre-miR-329, and mature miR-329-3p (B); pri-miR-494, pre-miR-494, and mature miR-494-3p (C); and pri-miR-495, pre-miR-495, and mature miR-495 (D).

mature miR-329-3p. The expression of pri-miR-495 and pre-miR-495 was too low to either confirm or exclude binding of CIRBP or HADHB. RIP experiments using an unrelated RBP that has been shown to regulate processing of polycistronic microRNAs, SND1,[29] showed no specific binding to either pri-miRNAs or pre-miRNAs of miR-329 and miR-495 (Figure S1).

Changes in MicroRNA Levels in CRISPR/Cas9-Generated HADHB KO Cell Lines

Next we sought to understand the effects depletion of CIRBP and HADHB would have on the levels of miR-329 and miR-495. To achieve this, we used CRISPR/Cas9 to generate 3T3 knockout (KO) cell lines. Our attempts to generate a CIRBP KO line were unsuccessful. This indicates that the protein could be essential for 3T3 cell vitality. Nevertheless, we identified two colonies (AQ and Z) grown from individual cells that had been targeted for disruption of HADHB. These colonies showed complete depletion of HADHB, as measured by western blot (Figure 6A). We confirmed this deletion by sequencing the region surrounding the targeted sequence (Figure 6B). All sequences identified either correspond to a frameshift deletion or a deletion spanning an exon-intron junction.

Following this, we analyzed the levels of mature miR-329 and miR-495 by qRT-PCR in colony AQ. We observed a decrease of over 80% in the levels of both mature miR-329-3p and miR-495-3p (Figure 6C). Importantly, the expression of miR-423, a widely expressed and stable microRNA not from the 14q32 cluster, did not change (Figure 6C). This suggests that the downregulation of mature miR-329-3p and miR-495-3p in HADHB KO cells is specific and not due to a global reduction in mature microRNA levels. Finally we determined the levels of pre-miR-329 and pre-miR-495 and showed lack of a statistically significant change (Figure 6D). Similar results were obtained for colony Z (data not shown). This corroborates our *in vivo* results and shows that HADHB is involved in posttranscriptional regulation of microRNAs from the 14q32 cluster at the level of pre-miRNA to mature microRNA cleavage.

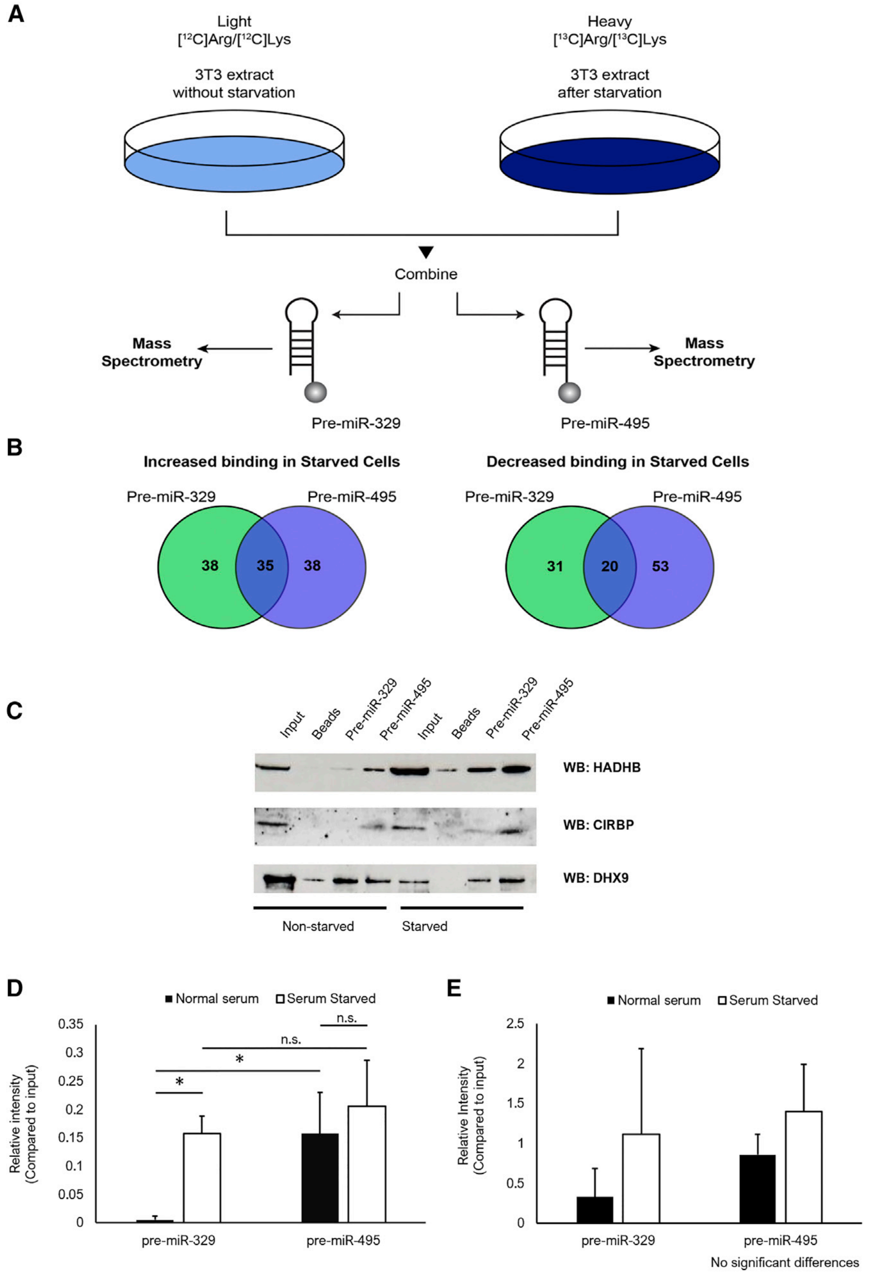

Figure 3. Identification of Proteins Binding Pre-miR-329 and Pre-miR-495

(A) Schematic representation of RNA pull-down combined with SILAC mass spectrometry. (B) Proteins showing increased or decreased binding to pre-miR-329, pre-miR-495, or both. (C) Western blot validation of HADHB and CIRBP binding to pre-miR-329 and pre-miR-495 under conditions of normal serum and serum starvation. DHX9 is used as a binding control. (D and E) Quantification of the western blot, showing HADHB (D) and CIRBP (E) binding to pre-miR-329 and pre-miR-495 under conditions of normal serum and serum starvation (relative to input). $^{*}p < 0.05$, n = 3.

DISCUSSION

In this study, we investigated the posttranscriptional regulation of 14q32 microRNAs during ischemia. Using specific primers for each of the microRNA processing steps, we found that increased expression of 14q32 microRNAs after hindlimb ischemia is not determined by increased transcription but that it is the result of increased processing of pre-miRNA to microRNA. We used serum starvation of 3T3 cells to mimic certain aspects of hindlimb ischemia. This allowed us to investigate which RBPs can bind to pre-miRNA transcripts of 14q32 microRNAs. Using RNA pull-down SILAC mass spectrometry, we found that CIRBP and HADHB bind to miR-329 and miR-495 precursors. We confirmed expression and upregulation of these RBPs in murine muscle tissues during ischemia at the mRNA level as well as the protein level for HADHB. Furthermore, we showed that, upon deletion of HADHB from 3T3 cells by CRISPR/Cas9, the levels of mature miR-329-3p and miR-495-3p, but not pre-miR-329 or pre-miR-495, are significantly reduced. These data are consistent with our *in vivo* data, supporting the hypothesis that HADHB is involved in the posttranscriptional regulation of these microRNAs in the pre-miRNA to mature microRNA processing step. Further in-depth analysis will be necessary to uncover the mechanism and physiological significance of HADHB- and CIRBP-mediated regulation of microRNA biogenesis.

Posttranscriptional regulation of polycistronic microRNAs has been described previously. In fact, differential expression after induction of hindlimb ischemia, similar to that of the 14q32 microRNAs, has also been shown for microRNAs of the polycistronic miR-17-92a cluster. The miR-17-92a cluster encodes seven microRNAs and is transcribed as a single primary transcript.[30] However, individual members of the miR-17-92a cluster are differentially expressed during endothelial differentiation of murine embryonic stem cells.[31] The SND1 protein, which is a component of the RISC, was found to bind to pri-miRNAs, pre-miRNAs, and mature microRNAs of the miR-17-92a cluster. Silencing of SND1 reduced processing of miR-17-92a cluster members, especially under hypoxic conditions.[29] Here we could not demonstrate binding of SND1 to either pri-miRNAs or pre-miRNAs of the 14q32 microRNAs miR-329 and miR-495 using RIP experiments. Our data demonstrated that processing of the 14q32 microRNAs miR-329 and miR-495

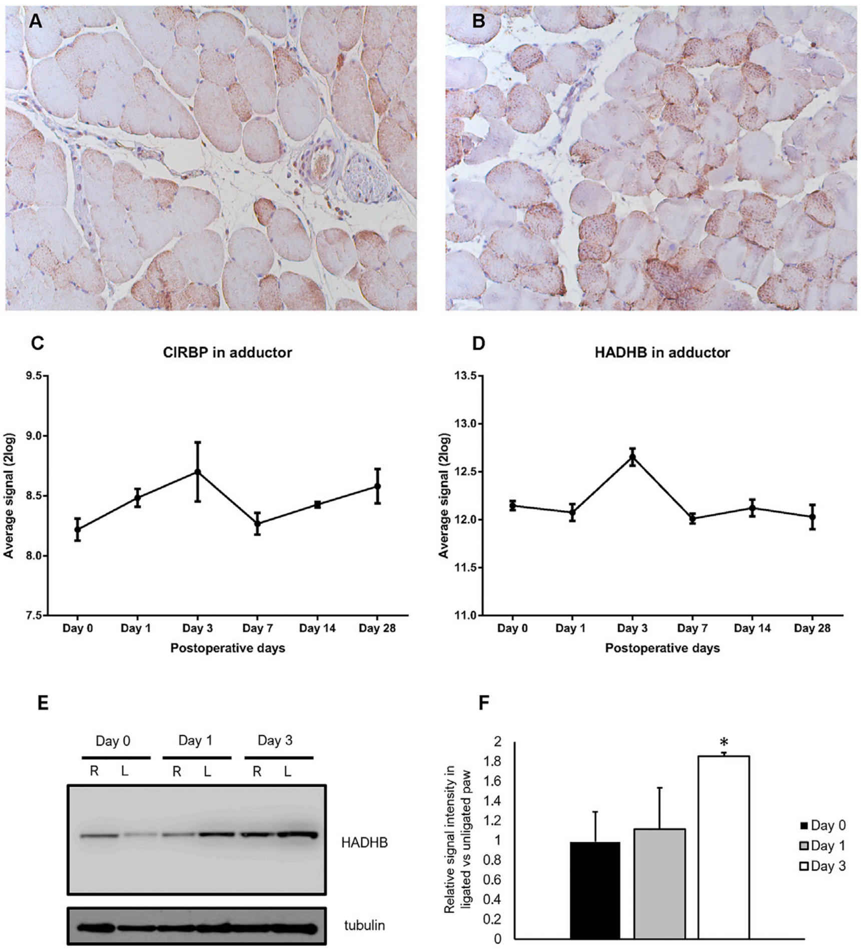

Figure 4. Expression of CIRBP and HADHB *In Vivo*

(A and B) Immunohistochemical staining of murine adductor muscle after ischemia induction revealed expression of both CIRBP (A) and HADHB (B) in these tissues, predominantly in the cytoplasm of cells. (C and D) Microarray analysis of mRNA expression of CIRBP (C) and HADHB (D) mRNA in the adductor muscle of mice at several time points after induction of ischemia (4 mice per time point). (E) Western blot showing HADHB levels in murine adductor muscle tissue on day 0, day 1, and day 3 after hindlimb ligation. For each time point, samples from right (R) unligated paws and left (L) ligated paws are presented next to each other. Tubulin was used as a loading control. (F) Quantification of the western blot. The HADHB signal was normalized against tubulin, and the relative change between ligated and unligated is presented. Each bar represents a biological triplicate and technical duplicate.

is independent of SND1 binding and instead relies on CIRBP and HADHB.

CIRBP is an evolutionarily conserved RBP that is transcriptionally upregulated under low-temperature conditions or other conditions of stress, including ischemia.[32,33] CIRBP protein is predominantly expressed in the nucleus but can also be transported to the cytoplasm under physiological or stressful conditions.[34] In our immunohistochemical stainings (Figures 4A and 4B), CIRBP and HADHB were observed in both the nucleus and the cytoplasm of intramuscular arteriolar wall cells. MicroRNA processing from pre- microRNA to mature microRNA also occurs in the cytoplasm. CIRBP is involved in posttranscriptional regulation of mRNAs.[35] Here, however, we showed for the first time that CIRBP could be involved in posttranscriptional regulation of microRNAs. Future experiments will have to determine whether CIRBP can also regulate other microRNAs during ischemia.

HADHB forms the β subunit of the mitochondrial trifunctional protein, which catalyzes the last steps of mitochondrial β-oxidation of long-chain fatty acids. In addition, HADHB has been found to act as an RBP and bind renin mRNA, leading to its destabilization.[36] Localization of HADHB has been found to be predominantly in mitochondria but also in the cytoplasm and nucleoli of Calu-6 cells.[37] In this study, we observed both cytoplasmic as well as nuclear expression of both HADHB and CIRBP in murine adductor muscle tissue after ischemia. In addition, we have now shown that HADHB can also bind pri-miRNAs, pre-miRNAs, and mature microRNAs of the 14q32 microRNA cluster, indicating its role in posttranscriptional regulation of microRNA expression under ischemia.

Regulation of microRNA processing under stress conditions such as hypoxia has been reported previously in several studies.[38–40] In endothelial cells, hypoxia has been shown to both increase the expression of certain microRNAs (such as miR-210[40]) as well as to reduce microRNA processing.[39] Further examination has revealed that chronic hypoxia downregulated the expression of Dicer, reducing subsequent microRNA processing.[39] More recently, the involvement of the epidermal growth factor receptor (EGFR) in microRNA processing under hypoxic conditions has been reported. EGFR has been shown to increase phosphorylation of AGO2 under hypoxic conditions, which reduced AGO2 binding to Dicer and subsequent microRNA processing from pre-miRNA to mature microRNA by Dicer.[27,41,42] Although we did not study the regulation of 14q32 microRNA processing under true hypoxic conditions *in vitro*, we were able to demonstrate increased binding of CIRBP and HADHB to pre-miRNAs under conditions of serum starvation, which is a major contributor to cellular stress after artery occlusion *in vivo*.

In this study, we identified RBPs that bind and regulate specific 14q32 microRNA precursors. These results provide insights into the complex regulation of the 14q32 microRNAs. We showed for the first time that CIRBP and HADHB, which have been shown to control

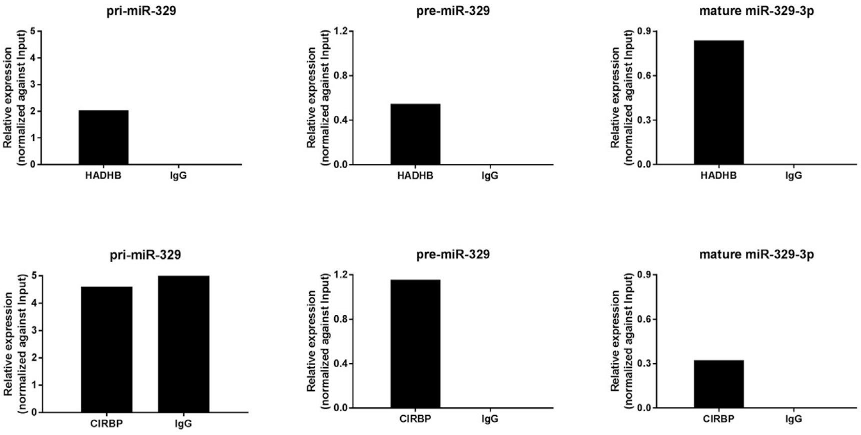

Figure 5. RNA Binding Protein Immunoprecipitation with HADHB and CIRBP Antibodies

Pri-miR-329, pre-miR-329, and mature miR-329 expression levels were measured in 3T3 cell lysates after immunoprecipitation with HADHB (top) and CIRBP (bottom) antibodies and non-specific IgG antibody. Bars represent technical triplicates.

posttranscriptional regulation of mRNAs, could also be involved in posttranscriptional processing of microRNAs. Finally, we demonstrated that depletion of HADHB in 3T3 cells has a negative effect on selected 14q32 microRNA expression levels. Through manipulation of CIRBP and HADHB, which control expression of 14q32 microRNAs, we may be able to influence 14q32 microRNA expression higher up in the regulatory cascade, potentially having more profound therapeutic effects on post-ischemic neovascularization. Because ischemic cardiovascular disease is still one of the leading causes of death worldwide, novel therapeutic options to induce neovascularization remain highly necessary.

MATERIALS AND METHODS

Hindlimb Ischemia Model

All experiments were approved by the Committee on Animal Welfare of the Leiden University Medical Center (Leiden, the Netherlands; approval reference numbers 09163 and 10243). This study was conducted in accordance with the Dutch government guidelines and Directive 2010/63/EU of the European Parliament. Unilateral hindlimb ischemia was induced in healthy adult male C57BL6 mice by single ligation of the left femoral artery, as described previously.[27,42] Briefly, electrocoagulation of the femoral artery was performed proximal to the superficial epigastric artery. C57BL/6 mice (n = 4 per time point) were sacrificed at several time points (day 0 [before ligation of the femoral artery] and days 1, 3, 7, 10, 14, and 28 after hindlimb ischemia induction).[43,44] Upon sacrifice, the adductor muscles were harvested and either snap-frozen on dry ice or fixed in 4% paraformaldehyde.

Microarray

For microarray analysis, total RNA was isolated from adductor muscles using the RNeasy Fibrous Tissue Minikit (QIAGEN). RNA concentration, purity, and integrity were analyzed by nanodrop (Nanodrop Technologies) and bioanalyzer (Agilent 2000) measurements.

For microRNA expression profiling, adductor muscle tissue from days 0, 1, 3, and 7 after induction of hindlimb ischemia was used. MicroRNA expression profiling was performed as described previously using locked nucleic acid (LNA)-based arrays (miRCURY LNA miR Array Ready-to-Spot Probe Set, Exiqon).[27] Normalization and background correction were performed in the "statistical language R" using the "vsn" package (Bioconductor). Differential expression was assayed using the "limma" package (Bioconductor) by fitting the eBayes linear model and contrasting individual treatments with untreated controls. Log2 fold changes were calculated using the toptable function of the limma package.[4,45]

For whole-genome expression profiling, adductor muscle tissue from days 0, 1, 3, 7, 14, and 28 after induction of hindlimb ischemia was used. Whole-genome expression profiling was performed using MouseWG-6 v2.0 Expression Beadchips (Illumina), and expression levels were Log2-transformed as described previously.[27,43]

Cell Culture

3T3 cells were cultured at 37°C in a humidified 5% CO_2 environment. The culture medium consisted of DMEM GlutaMAX (Gibco) supplemented with 10% heat-inactivated fetal calf serum (PAA Laboratories) and 1% penicillin and streptomycin (PAA). The culture medium was refreshed every 2–3 days. Cells were passed using trypsin-EDTA (Sigma) at 90% confluency.

In Vitro Cellular Starvation Model

To mimic the effects of nutrient depravation after *in vivo* ischemia, 3T3 cells were cultured under serum starvation conditions (DMEM GlutaMAX supplemented with 0.5% heat-inactivated fetal calf serum and 1% penicillin and streptomycin). Cells were starved for 24 hr under serum-starved conditions, after which they were collected and processed as necessary.

RNA Pull-down SILAC Mass Spectrometry

RNA pull-down and mass spectrometry (RP-SMS) experiments were performed as described previously with slight modifications.[46] In brief, total protein extracts from normal serum and serum-starved 3T3 cells grown in "light" [^{12}C]Arg/[12C]Lys and "heavy" [^{13}C]Arg/[^{13}C]Lys isotopes, respectively, were incubated with *in vitro*-transcribed RNAs chemically coupled to agarose beads. The incubation was followed by a series of washes with buffer G (20 mM Tris [pH 7.5], 135 mM NaCl, 1.5 mM MgCl2, 10% (v/v) glycerol, 1 mM EDTA, 1 mM DTT, and 0.2 mM PMSF). After the final wash, the proteins associated with the RNA on the beads were analyzed by SDS-PAGE followed by in-gel digestion and mass spectrometry or western blotting.

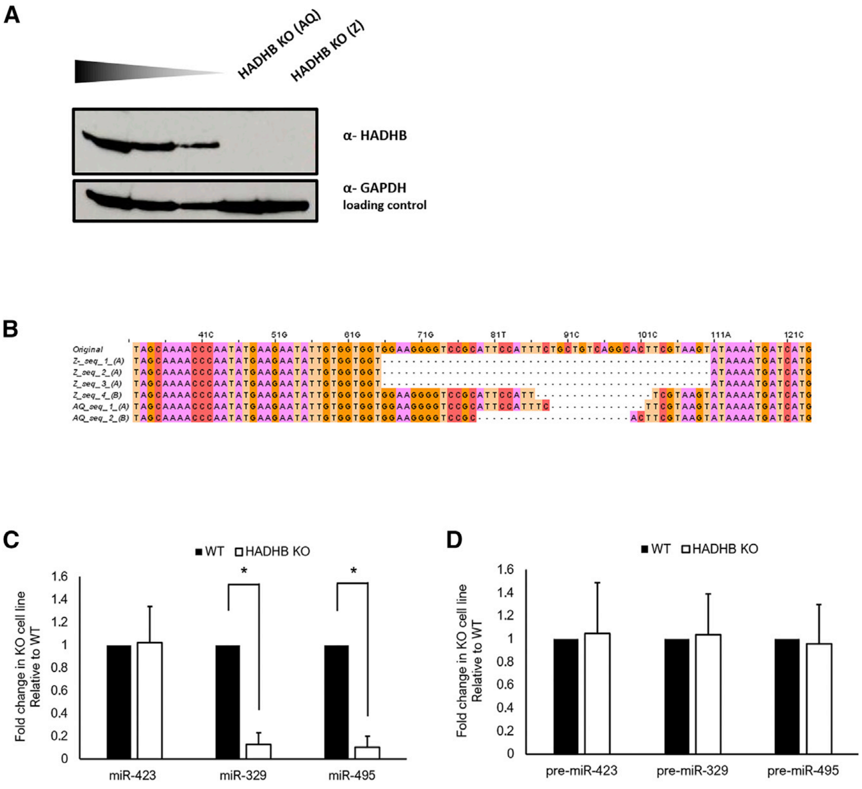

Figure 6. Generation of NIH 3T3 HADHB KO Cell Lines by CRISPR/Cas9

(A) Western blot analysis of HADHB protein levels in two HADHB KO clones (AQ and Z) compared with decreasing amounts of total protein extract from WT NIH 3T3 cells. (B) Alignment of the region surrounding the CRISPR-Cas9 target sequence from genomic DNA of clones AQ and Z. (C and D) Levels of mature (C) or precursor (D) miR-329 and miR-495 as well as control miR-423 in WT and HADHB KO cell lines quantified by qRT-PCR and normalized to miR-16. For pre-miRs, n = 3; for mature miRs, n = 9.

Western Blot Analysis

Total protein samples from 3T3 cells (100 μg per lane), isolated by sonication, were resolved by standard NuPAGE SDS-PAGE electrophoresis with MOPS running buffer (Life Technologies) and transferred onto a nitrocellulose membrane. Total protein samples from murine adductor muscle tissue were isolated using a standard TRIzol protocol (Thermo Fisher Scientific) on days 0, 1, and 3 after induction of ischemia. The membrane was blocked overnight at 4°C with 1:10 western blocking reagent (Roche) in Tris-buffered saline (TBS) buffer with 0.1% Tween 20 (TBST). The following day, the membrane was incubated for 1 hr at room temperature with primary antibody solution in 1:20 western blocking reagent diluted in TBST at the following concentrations: rabbit polyclonal CIRBP (ProteinTech, 10209-2-AP, 1:1,000) and rabbit polyclonal HADHB (LSBio-LS-C334236, 1:2,500). After washing in TBST, the blots were incubated with the appropriate secondary antibody conjugated to horseradish peroxidase and detected with SuperSignal West Pico detection reagent (Thermo Scientific). The membranes were stripped using ReBlot Plus Strong Antibody Stripping Solution (Chemicon) equilibrated in water, blocked in 1:10 western blocking solution in TBST, and re-probed as described above. Western blots were quantified using ImageJ analysis software (1.48v, NIH) and ImageStudio (LI-COR Biosciences) and normalized to the input.

RIP

RIP was performed using the EZMagna RIP kit (Millipore) according to the manufacturer's instructions. 3T3 cells were grown to 90% confluency and lysed in complete RIP lysis buffer. Cell lysates were incubated with RIP buffer containing magnetic beads conjugated with antibodies against CIRBP (Abcam, ab106230), HADHB (Novus Biologicals, NBP1-82609), SND1 (Abcam, ab71186), and rabbit control IgG (Millipore, PP64B). Before immunoprecipitation, 10% of cell lysate was taken and served as input control. Next, samples were treated with proteinase K to digest protein, and RNA was isolated using a standard TRIzol-chloroform extraction protocol.

qRT-PCR

Adductor muscles from day 0 and days 1, 3, and 7 after surgery were homogenized by grinding with a pestle and mortar in liquid nitrogen. Total RNA was isolated using a standard TRIzol-chloroform extraction protocol. RNA concentration and purity were determined by nanodrop (Nanodrop Technologies). RNA was reverse-transcribed using high-capacity RNA-to-cDNA RT kits (Life Technologies, USA). Relative quantitative mRNA PCR was performed on reverse-transcribed cDNA using SYBR green dye (QIAGEN). Primers for pri-miRNAs, pre-miRNAs, HADHB, and CIRBP were designed using Primer3. Sequences of primers are listed in Table S1. MicroRNA quantification was performed using TaqMan microRNA assays (Applied Biosystems) according to the manufacturer's protocol. Relative qPCR was performed on the Viia7 system (Applied Biosystems), and amplification efficiencies were checked by standard curves. Data were normalized using a stably expressed endogenous control (snRNA-U6).

Levels of mature miRs in 3T3 wild-type (WT) and HADHB KO cells were measured using miRScript RT (QIAGEN) and SYBR green (QIAGEN). The levels of pre-miRNAs were also measured by miRScript RT (QIAGEN) and SYBR green (QIAGEN) with prior fractionation of total RNA on 10% denaturing polyacrylamide gel to isolate the fraction containing RNAs from 50 nt to 100 nt. MicroRNA and pre-miRNA levels were normalized to miR-16 and pre-miR-16 because both proved to be highly stable in 3T3 cells.

Immunohistochemical Staining

Formaldehyde-fixed adductor muscles were paraffin-embedded, and 5-μm-thick cross-sections of muscles were stained to visualize the

expression of RBPs. Cross sections of adductor muscles were re-hydrated, and endogenous peroxidase activity was blocked. Antigen retrieval was performed with citrate buffer (pH 6.0) at 100°C for 10 min. Muscles were stained with rabbit polyclonal anti-HADHB (Novus Biologicals, NBP1-82609, 1:1,000) or goat polyclonal anti-CIRBP (Abcam, ab106230, 1:400) to visualize HADHB and CIRBP, respectively, and counterstained using hematoxylin.

Generation of HADHB KO by CRISPR/Cas9

3T3 cells were transfected with Cas9-containing plasmid px458, which included guide RNAs targeting the third exon of HADHB as well as a GFP cassette to be used as a reporter for positive transfection. Single-cell fluorescence-activated cell sorting (FACS) was carried out for selection of individual transfected cells, which were then grown into colonies from which genomic DNA was extracted. The region surrounding the Cas9 target was amplified by PCR and cloned into the pJET cloning vector (Thermo K1232) and sequenced using pJET sequencing primers. Western blotting was then used to confirm protein level depletion for colonies showing deletions in this region.

Statistical Analysis

Analysis of the microRNA and mRNA microarrays is described above.

Western blot and qRT-PCR data are represented as mean values ± SEM. Differences between groups were evaluated using one-way ANOVA followed by two-sample t test (Figures 3 and 4) or one-sample t test (Figure 6). $p > 0.05$ was considered statistically significant. The number of replicates is given in the figure legends.

SUPPLEMENTAL INFORMATION

Supplemental Information includes one figure and one table and can be found with this article online at https://doi.org/10.1016/j.omtn.2018.11.017.

AUTHOR CONTRIBUTIONS

Conceptualization, G.M. and A.Y.N.; Methodology, J.R., G.M., and A.Y.N.; Investigation, A.D.R.V., S.M.J.W., and E.A.C.G.; Writing – Original Draft, A.D.R.V., S.M.J.W., G.M., and A.Y.N.; Writing – Review & Editing, A.D.R.V., S.M.J.W., P.H.A.Q., G.M., and A.Y.N.; Funding Acquisition, J.R., G.M., P.H.A.Q., and A.Y.N.; Supervision, P.H.A.Q., G.M., and A.Y.N.

ACKNOWLEDGMENTS

A.D.R.V. was supported by CONACYT scholarship 684300 and a principal career development scholarship (Edinburgh). J.R. was supported by Wellcome Trust senior research fellowship 084229. G.M. was supported by Medical Research Council career development award G10000564, Wellcome Trust seed award 210144/Z/18/Z, and Wellcome Trust Centre core grants 077707 and 092076. A.Y.N. was supported by Netherlands Heart Foundation Dr. Dekker senior post-doctoral grant NHS-2014T102. S.M.J.W. and P.H.A.Q. were supported by NIRM (FES0908). We thank Christos Spanos for help with mass spectrometry.

REFERENCES

1. Bartel, D.P. (2004). MicroRNAs: genomics, biogenesis, mechanism, and function. Cell *116*, 281–297.
2. Schober, A., Nazari-Jahantigh, M., Wei, Y., Bidzhekov, K., Gremse, F., Grommes, J., Megens, R.T., Heyll, K., Noels, H., Hristov, M., et al. (2014). MicroRNA-126-5p promotes endothelial proliferation and limits atherosclerosis by suppressing Dlk1. Nat. Med. *20*, 368–376.
3. Yang, X., Du, W.W., Li, H., Liu, F., Khorshidi, A., Rutnam, Z.J., and Yang, B.B. (2013). Both mature miR-17-5p and passenger strand miR-17-3p target TIMP3 and induce prostate tumor growth and invasion. Nucleic Acids Res. *41*, 9688–9704.
4. Lu, M., Zhang, Q., Deng, M., Miao, J., Guo, Y., Gao, W., and Cui, Q. (2008). An analysis of human microRNA and disease associations. PLoS ONE *3*, e3420.
5. Lujambio, A., and Lowe, S.W. (2012). The microcosmos of cancer. Nature *482*, 347–355.
6. Im, H.I., and Kenny, P.J. (2012). MicroRNAs in neuronal function and dysfunction. Trends Neurosci. *35*, 325–334.
7. Ono, K., Kuwabara, Y., and Han, J. (2011). MicroRNAs and cardiovascular diseases. FEBS J. *278*, 1619–1633.
8. Welten, S.M., Goossens, E.A., Quax, P.H., and Nossent, A.Y. (2016). The multifactorial nature of microRNAs in vascular remodelling. Cardiovasc. Res. *110*, 6–22.
9. Boon, R.A., and Dimmeler, S. (2015). MicroRNAs in myocardial infarction. Nat. Rev. Cardiol. *12*, 135–142.
10. Suzuki, H.I., Yamagata, K., Sugimoto, K., Iwamoto, T., Kato, S., and Miyazono, K. (2009). Modulation of microRNA processing by p53. Nature *460*, 529–533.
11. Choudhury, N.R., de Lima Alves, F., de Andrés-Aguayo, L., Graf, T., Cáceres, J.F., Rappsilber, J., and Michlewski, G. (2013). Tissue-specific control of brain-enriched miR-7 biogenesis. Genes Dev. *27*, 24–38.
12. Michlewski, G., Guil, S., Semple, C.A., and Cáceres, J.F. (2008). Posttranscriptional regulation of miRNAs harboring conserved terminal loops. Mol. Cell *32*, 383–393.
13. Guil, S., and Cáceres, J.F. (2007). The multifunctional RNA-binding protein hnRNP A1 is required for processing of miR-18a. Nat. Struct. Mol. Biol. *14*, 591–596.
14. Davis, B.N., Hilyard, A.C., Lagna, G., and Hata, A. (2008). SMAD proteins control DROSHA-mediated microRNA maturation. Nature *454*, 56–61.
15. Sakamoto, S., Aoki, K., Higuchi, T., Todaka, H., Morisawa, K., Tamaki, N., Hatano, E., Fukushima, A., Taniguchi, T., and Agata, Y. (2009). The NF90-NF45 complex functions as a negative regulator in the microRNA processing pathway. Mol. Cell. Biol. *29*, 3754–3769.
16. Wu, H., Sun, S., Tu, K., Gao, Y., Xie, B., Krainer, A.R., and Zhu, J. (2010). A splicing-independent function of SF2/ASF in microRNA processing. Mol. Cell *38*, 67–77.
17. Trabucchi, M., Briata, P., Garcia-Mayoral, M., Haase, A.D., Filipowicz, W., Ramos, A., Gherzi, R., and Rosenfeld, M.G. (2009). The RNA-binding protein KSRP promotes the biogenesis of a subset of microRNAs. Nature *459*, 1010–1014.
18. Michlewski, G., and Caceres, J.F. (2018). Post-transcriptional control of miRNA biogenesis. RNA. Published online October 17, 2018. https://doi.org/10.1261/rna.068692.118.
19. Viswanathan, S.R., Daley, G.Q., and Gregory, R.I. (2008). Selective blockade of microRNA processing by Lin28. Science *320*, 97–100.
20. Michlewski, G., and Cáceres, J.F. (2010). Antagonistic role of hnRNP A1 and KSRP in the regulation of let-7a biogenesis. Nat. Struct. Mol. Biol. *17*, 1011–1018.
21. Newman, M.A., Thomson, J.M., and Hammond, S.M. (2008). Lin-28 interaction with the Let-7 precursor loop mediates regulated microRNA processing. RNA *14*, 1539–1549.
22. Piskounova, E., Polytarchou, C., Thornton, J.E., LaPierre, R.J., Pothoulakis, C., Hagan, J.P., Iliopoulos, D., and Gregory, R.I. (2011). Lin28A and Lin28B inhibit let-7 microRNA biogenesis by distinct mechanisms. Cell *147*, 1066–1079.
23. Choudhury, N.R., Nowak, J.S., Zuo, J., Rappsilber, J., Spoel, S.H., and Michlewski, G. (2014). Trim25 Is an RNA-Specific Activator of Lin28a/TuT4-Mediated Uridylation. Cell Rep. *9*, 1265–1272.

24. Heo, I., Joo, C., Kim, Y.K., Ha, M., Yoon, M.J., Cho, J., Yeom, K.H., Han, J., and Kim, V.N. (2009). TUT4 in concert with Lin28 suppresses microRNA biogenesis through pre-microRNA uridylation. Cell *138*, 696–708.

25. Heo, I., Joo, C., Cho, J., Ha, M., Han, J., and Kim, V.N. (2008). Lin28 mediates the terminal uridylation of let-7 precursor MicroRNA. Mol. Cell *32*, 276–284.

26. Rybak, A., Fuchs, H., Smirnova, L., Brandt, C., Pohl, E.E., Nitsch, R., and Wulczyn, F.G. (2008). A feedback loop comprising lin-28 and let-7 controls pre-let-7 maturation during neural stem-cell commitment. Nat. Cell Biol. *10*, 987–993.

27. Welten, S.M., Bastiaansen, A.J., de Jong, R.C., de Vries, M.R., Peters, E.A., Boonstra, M.C., Sheikh, S.P., La Monica, N., Kandimalla, E.R., Quax, P.H., and Nossent, A.Y. (2014). Inhibition of 14q32 MicroRNAs miR-329, miR-487b, miR-494 and miR-495 Increases Neovascularization and Blood Flow Recovery after Ischemia. Circ Res. *115*, 696–708.

28. Welten, S.M.J., de Vries, M.R., Peters, E.A.B., Agrawal, S., Quax, P.H.A., and Nossent, A.Y. (2017). Inhibition of Mef2a Enhances Neovascularization via Post-transcriptional Regulation of 14q32 MicroRNAs miR-329 and miR-494. Mol. Ther. Nucleic Acids *7*, 61–70.

29. Heinrich, E.M., Wagner, J., Krüger, M., John, D., Uchida, S., Weigand, J.E., Suess, B., and Dimmeler, S. (2013). Regulation of miR-17-92a cluster processing by the microRNA binding protein SND1. FEBS Lett. *587*, 2405–2411.

30. He, L., Thomson, J.M., Hemann, M.T., Hernando-Monge, E., Mu, D., Goodson, S., Powers, S., Cordon-Cardo, C., Lowe, S.W., Hannon, G.J., and Hammond, S.M. (2005). A microRNA polycistron as a potential human oncogene. Nature *435*, 828–833.

31. Tréguer, K., Heinrich, E.M., Ohtani, K., Bonauer, A., and Dimmeler, S. (2012). Role of the microRNA-17-92 cluster in the endothelial differentiation of stem cells. J. Vasc. Res. *49*, 447–460.

32. Wellmann, S., Bührer, C., Moderegger, E., Zelmer, A., Kirschner, R., Koehne, P., Fujita, J., and Seeger, K. (2004). Oxygen-regulated expression of the RNA-binding proteins RBM3 and CIRP by a HIF-1-independent mechanism. J. Cell Sci. *117*, 1785–1794.

33. Jackson, T.C., Manole, M.D., Kotermanski, S.E., Jackson, E.K., Clark, R.S., and Kochanek, P.M. (2015). Cold stress protein RBM3 responds to temperature change in an ultra-sensitive manner in young neurons. Neuroscience *305*, 268–278.

34. Zhu, X., Bührer, C., and Wellmann, S. (2016). Cold-inducible proteins CIRP and RBM3, a unique couple with activities far beyond the cold. Cell. Mol. Life Sci. *73*, 3839–3859.

35. Lleonart, M.E. (2010). A new generation of proto-oncogenes: cold-inducible RNA binding proteins. Biochim. Biophys. Acta *1805*, 43–52.

36. Adams, D.J., Beveridge, D.J., van der Weyden, L., Mangs, H., Leedman, P.J., and Morris, B.J. (2003). HADHB, HuR, and CP1 bind to the distal 3′-untranslated region of human renin mRNA and differentially modulate renin expression. J. Biol. Chem. *278*, 44894–44903.

37. Morris, B.J., Adams, D.J., Beveridge, D.J., van der Weyden, L., Mangs, H., and Leedman, P.J. (2004). cAMP controls human renin mRNA stability via specific RNA-binding proteins. Acta Physiol. Scand. *181*, 369–373.

38. Leung, A.K., and Sharp, P.A. (2010). MicroRNA functions in stress responses. Mol. Cell *40*, 205–215.

39. Ho, J.J., Metcalf, J.L., Yan, M.S., Turgeon, P.J., Wang, J.J., Chalsev, M., Petruzziello-Pellegrini, T.N., Tsui, A.K., He, J.Z., Dhamko, H., et al. (2012). Functional importance of Dicer protein in the adaptive cellular response to hypoxia. J. Biol. Chem. *287*, 29003–29020.

40. Fasanaro, P., D'Alessandra, Y., Di Stefano, V., Melchionna, R., Romani, S., Pompilio, G., Capogrossi, M.C., and Martelli, F. (2008). MicroRNA-210 modulates endothelial cell response to hypoxia and inhibits the receptor tyrosine kinase ligand Ephrin-A3. J. Biol. Chem. *283*, 15878–15883.

41. Shen, J., Xia, W., Khotskaya, Y.B., Huo, L., Nakanishi, K., Lim, S.O., Du, Y., Wang, Y., Chang, W.C., Chen, C.H., et al. (2013). EGFR modulates microRNA maturation in response to hypoxia through phosphorylation of AGO2. Nature *497*, 383–387.

42. Nowak-Sliwinska, P., Alitalo, K., Allen, E., Anisimov, A., Aplin, A.C., Auerbach, R., Augustin, H.G., Bates, D.O., van Beijnum, J.R., Bender, R.H.F., et al. (2018). Consensus guidelines for the use and interpretation of angiogenesis assays. Angiogenesis. Published online May 15, 2018.. https://doi.org/10.1007/s10456-018-9613-x

43. Nossent, A.Y., Bastiaansen, A.J., Peters, E.A., de Vries, M.R., Aref, Z., Welten, S.M., de Jager, S.C., van der Pouw Kraan, T.C., and Quax, P.H. (2017). CCR7-CCL19/CCL21 Axis is Essential for Effective Arteriogenesis in a Murine Model of Hindlimb Ischemia. J. Am. Heart Assoc. *6*, e005281.

44. Simons, K.H., Aref, Z., Peters, H.A.B., Welten, S.P., Nossent, A.Y., Jukema, J.W., Hamming, J.F., Arens, R., de Vries, M.R., and Quax, P.H.A. (2018). The role of CD27-CD70-mediated T cell co-stimulation in vasculogenesis, arteriogenesis and angiogenesis. Int. J. Cardiol. *260*, 184–190.

45. van der Kwast, R.V., van Ingen, E., Parma, L., Peters, H.A., Quax, P.H., and Nossent, A.Y. (2017). A-To-I Editing of Microrna-487b Alters Target Gene Selection After Ischemia and Promotes Neovascularization. Circ. Res. *122*, 444–456.

46. Choudhury, N.R., and Michlewski, G. (2018). Quantitative identification of proteins that influence miRNA biogenesis by RNA pull-down–SILAC mass spectrometry (RP-SMS). Methods. Published online June 8, 2018. https://doi.org/10.1016/j.ymeth.2018.06.006.

OMTN, Volume *14*

Supplemental Information

Posttranscriptional Regulation of 14q32 MicroRNAs by the CIRBP and HADHB during Vascular Regeneration after Ischemia

Angela Downie Ruiz Velasco, Sabine M.J. Welten, Eveline A.C. Goossens, Paul H.A. Quax, Juri Rappsilber, Gracjan Michlewski, and A. Yaël Nossent

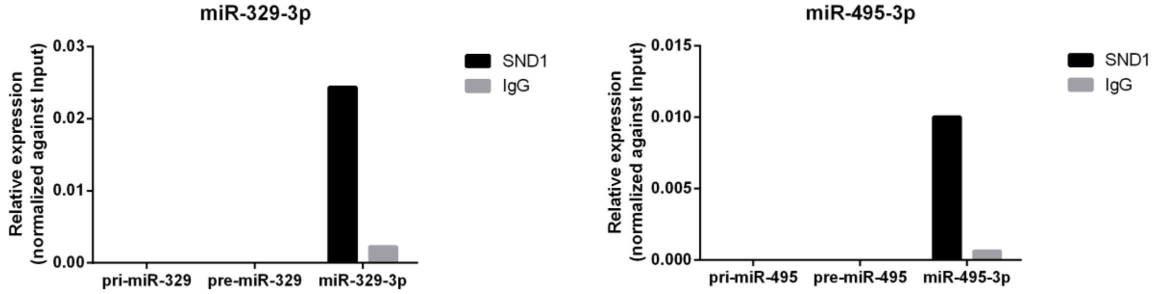

Supplemental Figure 1. RNA binding protein immunoprecipitation with SND1 antibody. Pri-miR, pre-miR and mature microRNA expression levels of miR-329-3p and miR-495-3p were measured in 3T3 cell lysates after immunoprecipitation with SND1 antibody and a non-specific IgG antibody.

Supplementary Table 1. List of primers used.

Gene	Forward Primer	Reverse Primer
primiR-329	AAGGTCACGTTGGGGAATTA	ACCACGAAGCCTCCAAGAT
premiR-329	TGGTACCGGAAGAGAGGTTTT	AGGTTAGCTGGGTGTGTTTCA
primiR-495	AGCATCCCTTCACACTCAGG	GAGCTCTCCAAGGTGAGATTTG
premiR-495	GTTGCCCATGTTATTTTTCG	AGTGCACCATGTTTGTTTCG
CIRBP	TTTTCGTGGGAGGACTCAGC	CCCTGTCCTTTACCACCACC
HADHB	CAGCGCCTGTCCTTACTCAG	CAGAGTGGCCCATGGTCTC
